# An Indian Civil Medical Service

**Published:** 1918-06-08

**Authors:** 


### AN INDIAN CIVIL MEDICAL SERVICE.

A report of the session of the Indian Imperial Legislative Council is in the *Morning Post* of May 23, 1918. A part of the proceedings dealt with the Indian Medical Service, the discussion arising on the Report of the Public Services Commission. Mr. Sastri proposed that a Civil Medical Service should be constituted which should be wholly independent of the medical organisation of the Indian Army. This proposal is not new. It has, in fact, often been put forward, the idea usually formulated being that the R.A.M.C. should take over the medical treatment of the Indian Army, that the regimental medical officers of Indian regiments should be abolished, and the station hospital organisation, suitably modified, be brought in for the treatment of the sick native soldier.

It is not proposed to dogmatise on the desirability of the change. The Indian Native Army differs much from a European army, so what suits one does not necessarily suit the other. The regimental medical officer of an Indian regiment is seldom with his regiment. He who shows as the medical officer in the Army List as often as not is doing other than regimental work, and some officer junior to him is in temporary charge. That this is so is evidence favouring the conclusion that the appointment of a permanent medical officer to a regiment is not necessary. In fact, the greater part of the best of Indian medical officers only do regimental duty till they can get a billet on the civil side. This is not because the Army regimental work is unpleasant, but because the civil work gives better professional opportunities and better pay than the regimental work. The practice obtainable by the regimental medical officer is very small. That is one of the arguments in favour of a station hospital system. The great improvement in professional knowledge and skill that has marked the change from a regimental system to the corps system in the British Army would extend, it is held, to the Army branch of the Indian Medical Service, or, more correctly, if the men who treat the Indian soldier were all members of the R.A.M.C., and if the treatment of the Indian soldier were carried out in station hospitals, the men of the larger R.A.M.C. would gain by the more varied experience, the Indian soldier would gain by better treatment. The details of organisation cannot be gone into in the space of a short article.

But it is doubtful if zeal for the advancement of medical knowledge, or for the better treatment of the Indian soldier, has moved Mr. Sastri to his proposal. It is pretty obvious from the context that jealousy of the European element in the Indian Medical Service—a trade union spirit, not a reforming spirit, is at the bottom of it. The Medical Civil Service is to be kept for Indians quite regardless of the way such policy may affect the millions handed over to the care of the new Service, or of how it may affect the progress of scientific knowledge in India. Such seems to be the reading between the lines; and if it be, it behoves the peoples of all the hundred nations, and all the hundred religions, of India to beware of the proposals.

